# Retrospective Review on Accuracy: A Pilot Study of Robotically Guided Thoracolumbar/Sacral Pedicle Screws Versus Fluoroscopy-Guided and Computerized Tomography Stealth-Guided Screws

**DOI:** 10.7759/cureus.1437

**Published:** 2017-07-06

**Authors:** Brian Fiani, Syed A Quadri, Vivek Ramakrishnan, Blake Berman, Yasir Khan, Javed Siddiqi

**Affiliations:** 1 Institute of Clinical Orthopedic and Neurosciences (Icon), Desert Regional Medical Center, Palm Springs, Ca; 2 Neurosurgery, California Institute of Neurosciences

**Keywords:** pedicle, pedicle screw, screws, robotic, hand-placed, spinal instrumentation, spine, accuracy, fluoroscopy, computerized tomography (ct)

## Abstract

Introduction

Pedicle screw insertion is the mainstay of thora­cic and lumbosacral posterior spinal instrumentation. However, it may be associated with complications such as screw mal­positioning. The purpose of this study was to develop a pilot study to compare the accuracy of robot-guided screw insertion versus hand-guided screw placement for spinal instrumentation. The hand-guided screws were placed with assistance from computerized tomography (CT) stealth guidance or fluoroscopy.

Materials and methods

A retrospective analysis of medical records was done for all patients that had pedicle screw insertion for instrumentation between the dates of December 2013 and January 2016 with post-screw placement CT imaging. The analysis was conducted on screw accuracy between the two categories based on the Gertzbein-Robbins classification.

Results

A total of 49 screws were analyzed for accuracy in six patients. There was no statistically significant difference between the accuracy of hand-placed pedicle screws versus the robotically placed screws (p = 0.311). There was no statistically significant difference in blood loss (p = 0.616), length of procedure (p = 0.192), or post-operative length of stay (p = 0.587).

Conclusion

The findings of our pilot study agree with most prior studies that there was no statistically significant difference in the accuracy of pedicle screw placement between the two methods of screw placement. Therefore, the techniques are equivocal in accuracy. The new technology (robotic-guidance) is as safe as conventional techniques for screw placement. Just like in any surgery, the technique preference should remain surgeon dependent. The results are only from a small sample size in the development of a pilot study so a strong reliance on the data would not be suggested. The study was a preliminary study that will be used as a template and learning process to create a future prospective study to investigate CT stealth and robotically guided screw placement versus “free hand” guided screws.

## Introduction

Pedicle screw insertion of the spine is one of the main procedural steps of thora­cic and lumbosacral posterior instrumentation and has gone through substantial advancement over the last couple of decades [1-2]. An array of pedicle screw systems has been described and new systems are being developed every day. The technique and principles of screw placement, as well as, anatomical landmarks of screw placement, however, are common to all systems. However, pedicle screw placement may be associated with complications such as screw malpositioning [2-3].

In recent years, image guidance with navigation and intraoperative imaging have been used in order to improve the accuracy and safety of pedicle screw placement. In addition to these navigational systems, a robotic spine surgery system has also been recently added to the arsenal in order to increase the accuracy of pedicle screw trajectories [4-6]. While Ringel, et al. claimed that the accuracy of the conventional free-hand technique was superior to the robot-assisted technique some studies have found more clinically acceptable screw placements with robotic spine surgery [3, 7-8]. Hence, the question of precision of screw placement between the two methods still remains unanswered to date.

The main purpose of this pilot study is to compare the accuracy of robot-guided screw insertion in thoracolumbar and sacral surgeries at our center with a cohort of patients who underwent hand-guided screw placement via fluoroscopy-guided or computed tomography (CT) stealth-guided spinal instrumentation. It is hypothesized that accuracies will remain similar between the two cohorts without a significant difference, making the robotic technique equivocally safe to use in spinal surgery. The secondary purpose was to use this pilot study to design a prospective multicenter study with a much larger cohort to validate the findings of present and prior studies and the question regarding screw placement precision superiority.

## Materials and methods

Patients

A retrospective analysis of medical records was performed for all patients that had pedicle screw insertion for instrumentation between December 2013 and January 2016 with post-screw placement CT imaging. The data was extracted via Crimson Continuum of Care system at Desert Regional Medical Center, in Palm Springs, California. The patients who were over 18 years of age and underwent spinal fusion surgery, except cervical spine, and having post-operative CT scan were included.

Robot and implants

Bone-mounted or table-mounted, miniature robotic spine surgery system named the Renaissance system by Mazor was used.

Assessment of screw position

The primary objective was to compare the accuracy of screw placement with fluoroscopy-guided hand-placed screw versus robotic-placed screws. This was done by measuring the screw accuracy with the Gertzbein and Robbins Scale as described below [9]. A blinded investigator with neurosurgical training interpreted the post-operative CT scans to assess the accuracy of screw placement by using the scale. The investigator, who was blinded to the insertion technique used by the neurosurgeon, analyzed all CT’s in both the sagittal and axial perspectives.

Gertzbein and Robbins scale

The Gertzbein and Robbins scale is one of two grading scales currently used to describe pedicle screw placement [9]. In this system, Grade A screws are those that are fully contained within a pedicle with no evidence of cortical breach, while higher grades are assigned in breach distances of multiples of 2 mm, where distance is measured from the medial, lateral, superior, or inferior border of the pedicle (Table 1). This scale was first applied when assessing screws placed from T8 to S1. Grade A screws do not show evidence of pedicle breach, Grade B screws breach 0 mm to 2 mm, and Grade C screws are those that breached 2 mm to 4 mm. According to Gertzbein and Robbins, a 4-mm "safe zone" exists in the lumbar region adjacent to the pedicle for screws placed from T10 to L4 and a satisfactory outcome without clinical neurologic complications can be observed for screws violating the pedicle by 0 to 4 mm. Grade D was assigned to screws with 4-6 mm of breach. Lastly, Grade E was given to screws with >6 mm of cortical wall breach.

**Table 1:** Gertzbein-Robbins classification of pedicle screw accuracy.

Gertzbein and Robbins scale	
Grade	Breach distance
A	0 mm (no breach)
B	0-2 mm
C	2-4 mm
D	4-6 mm
E	>6 mm

Collection of other clinical data

Other parameters that were recorded from the patient charts included operative time, blood loss, and hospital length of stay (LOS) from the day of surgery. The analysis was based on intraoperative and postoperative medical documentation. All information was extracted from the patients’ charts and CT scans.

Statistical analysis

The Pearson Correlation test was used for statistical analysis.

## Results

There were in total six patients who underwent pedicle screw insertion for thoracolumbar/sacral pathology or trauma during the aforementioned period. Four patients had fluoroscopy-guided or CT stealth-guided hand-placed screws and two patients had robotically placed screws. Average patient age was 52.5 years in the robotic group (Group A) and 59.5 years in the hand-placed group (Group B). Patient demographic has been summarized in the table (Table 2).

**Table 2:** Overview of patient characteristics and demographic in Group A and Group B. BMI: Body mass index.

Demographics	Robotically placed (Group A)	Hand placed (Group B)
Number of patients	2	4
Sex (Male:Female)	(1:1)	(1:1)
Age	52.5	59.5
Height	171.45	170.75
Weight	77.0915	90
Race (Hispanic:Black:White:Other)	(0:0:2:0)	(0:0:4:0)
BMI	26.27611004	30.80321734
Number of screws	16	33

**Figure 1:**
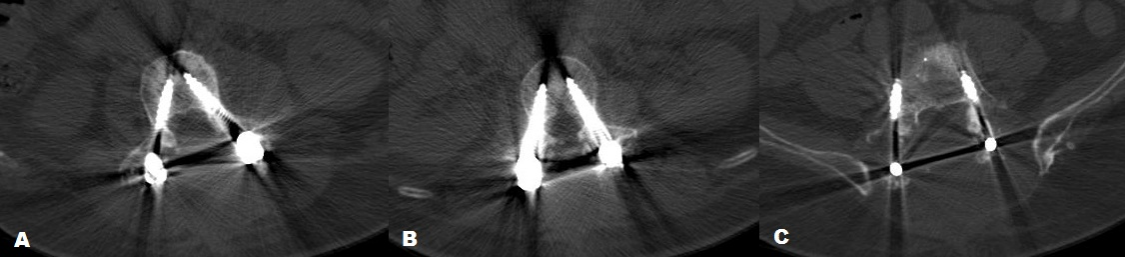
Computed tomography (CT) scan of fluoroscopy-guided hand-placed screws (A) demonstrates Grade-A accuracy for screw placement at L3 level (B) demonstrates Grade-B accuracy at L2 level. (C) Right screw demonstrates Grade-C placement and left shows Grade-B placements at level L5 according to Gertzbein-Robbins classification.

**Figure 2:**
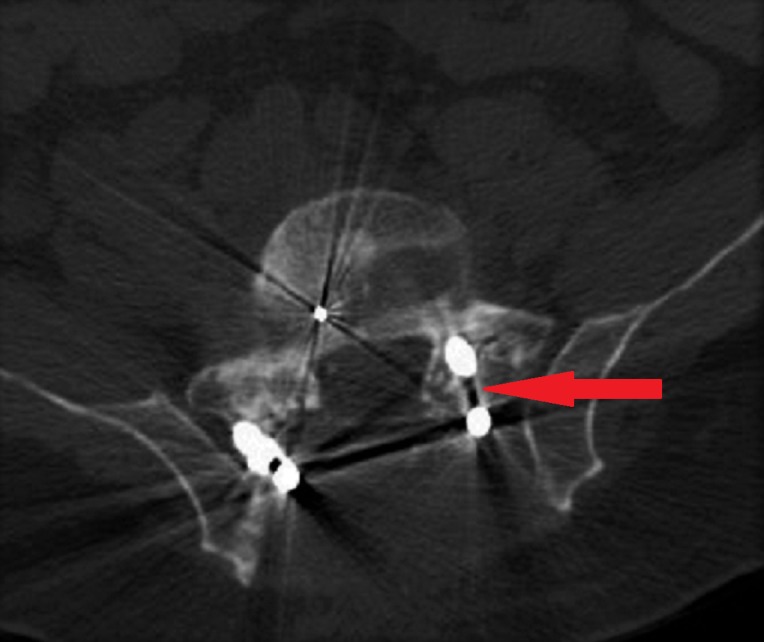
Computed tomography (CT) scan of fluoroscopy-guided hand-placed screws demonstrates the only Grade-E accuracy for screw placement (Right screw) as denoted by arrow at S1 level.

**Figure 3:**
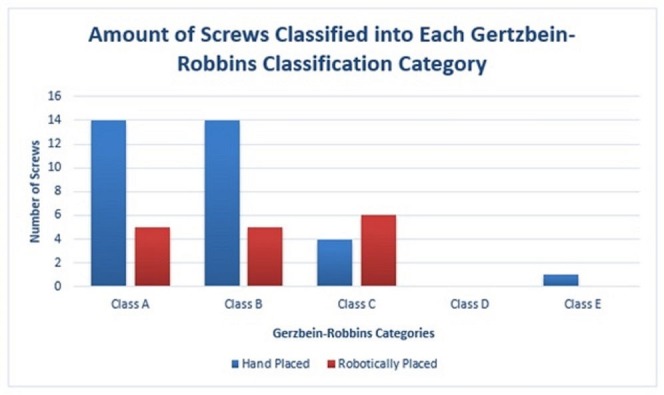
A bar graph representation of the amount of screws classified in each Gertzbein-Robbins classification category.

A total of 49 screws were placed which were analyzed for accuracy. Thirty-three (67.3%) were in the hand-placed group (Group B) (Figure 1) and 16 (32.6%) were in the robotically placed group (Group A). According to the Gertzbein and Robbins scale for Grade A (0 mm), there were 14 hand-placed (42.42%) and five robotically placed (31.25%) screws. For Grade B (<2 mm), there were 14 hand-placed (42.42%) and five robotically placed (31.25%) screws. For Grade C (2-4 mm), there were four hand-placed (12.12%) and six robotically placed (31.25%) screws. There were no screws having Grade D (4-6 mm). For Grade E (>6 mm), there was only one hand-placed screw (3.03%) (Figure 2). The amount of screws classified in each category is represented by bar graph (Figure 3). There was no statistically significant difference between the accuracy of hand-placed pedicle screws versus the robotically placed screws (p = 0.311). The frequency of accuracy for each screw placed in either group is shown in Table 3.

There was no statistically significant difference in blood loss (p = 0.616) with an average blood loss of 1625 ml for hand placed and 975 ml blood loss for robotic (Table 3). The duration/length of procedure documented in the patients’ records included all surgical steps from initial incision to wound closure. The average length of procedure for the hand-placed group was 407.5 mins and for the robotic group was 315 mins (p = 0.192). The average post-operative length of stay for the hand-placed group was 9 days and 4.5 for the robotic group (p = 0.587).

**Table 3:** Overview of other parameters recorded for each patient in Group A and Group B.

Type of surgery planned: Robot-guided technique (Group A), Hand-guided surgery (Group B)	Blood loss (ml)	Total procedure time (min)	Length of hospital stay (LOS) post-procedure (Days)
B	700	376	3
B	4000	476	6
A	1200	335	6
A	750	259	3
B	900	482	24
B	900	296	3

## Discussion

As pedicle screw fixation was first described by Boucher in the 1950s, used more extensively and further examined by Roy-Camille later in the 1960s and 1970s, and then down-classified from an FDA Class III to Class II device in 1998, pedicle screw fixation has evolved and become increasingly popular among spine surgeons [10-13]. Originally, pedicle screws were used primarily in the lumbar spine, but as the surgeons have become more comfortable with the intricate anatomy required for accurate screw placement, the technique for pedicle instrumentation has evolved to include their use in the thoracolumbar and thoracic spinal levels as well [14-15].

In addition to offering an overall increased construct rigidity, the pedicle screws have several advantages over the traditional hook and rod constructs as they allow the stability essential for spinal arthrodesis and improve deformity correction due to its three-column control over the spinal elements. Pedicle screw fixation promotes multidimensional control and may provide greater fusion rates, making it the mainstay of thoracic and lumbosacral posterior spine instrumentation [2, 7, 16-18]. However, it may be associated with complications such as malpositioning which may lead to possible nerve root injury or superior facet joint violation [2-3]. Intraoperative fluoroscopic imaging and image-guided navigation are some of the developments in recent years that have been implemented in efforts to improve the accuracy, and subsequently, the safety of pedicle screw placement.

Robotics, already adopted in other surgical specialties, has also become an innovative development in spine surgery to further improve accuracy and safety [4-6, 19-22]. There are two robot designs available for spine surgery, the first being a supervisory-controlled system and the second being a master-slave system. Supervisory-controlled systems, like the Mazor robot used in this study, reproduce movements that the robot was previously instructed to perform. This type of system is helpful in guiding the trajectory for biopsy, kyphoplasty, or as in this study, pedicle screw placement. The trajectory is planned in accordance to pre-operative imaging and can also be manipulated intraoperatively on the robotic console. Master-slave systems, however, allow surgeons to directly translate their movements in real-time through a console.

In the past, there have been studies comparing robot-assisted placed screws with fluoroscopy-guided hand placed screws with mix results [23-28]. There have been studies showing either of the two to be more accurate while others showed no difference [23-28]. Schatlo, et al., Kantelhardt, et al. and Pechlivanis, et al. verified the increased overall accuracy with robotically-assisted screw placement while Schizas, et al. and Lieberman, et al. reported less misplacement using the new robotic technology [3, 24-27]. On the other hand, Ringel, et al. found the conventional free-hand technique to be more superior in terms of accuracy [23]. The question of precision of screw placement between the two methods still remains unanswered to date. The intent of study was to evaluate and validate which method was more accurate using the Gertzbein and Robbins scale. The null hypothesis was that there would be no difference in the accuracy of the screws placed via either method.

In this study, six patients were evaluated having 49 screws inserted in total. Out of these, 33 screws were inserted by hand placement and 16 were robotically guided. The accuracy of pedicle screw placement in cohorts of robotic-guided and conventionally placed pedicle screws was assessed. Assessment of pedicle screw position by a single investigator blinded to the insertion technique was performed in order to minimalize the effect of investigator-dependent errors. The investigator also is a neurosurgery trained doctor with experience in image analysis. The long-term results (fusion rate, etc.) were not included in our study as it can be anticipated that these would be similar for all pedicle screws regardless of the surgical approach applied. Even more difficult would be finding patients that received serial follow-up imaging post-operatively. The finding of this study showed that there is no statistically significant difference between the accuracy of the two methods for the guidance of pedicle screw placements (p = 0.311).

Pechlivanis, et al. and Kantelhardt, et al. reported 98.5% and 98.9% accuracy (Grade A and B) with robot-assisted group [24, 26]. Schatlo, et al. also reported 91.4% accuracy (Grade A and B) in robot-assisted cohort [3]. In our study, the accuracy (Grade A and B) with robot-assisted group was 62.5%. On the other hand, Ringel, et al. reported 93% good positions (A or B) with fluoroscopy-guided or CT stealth-guided free hand technique compared to 85% with robotic screw placement and found the free hand technique to be more superior [23]. Due to the small number of study cohorts and the ratio between the hand placed and robotic groups being (2:1), the p-value calculated for accuracy of hand-placed pedicle screws versus the robotically placed screws (p = 0.311) in our study is not of statistical significance as compared to the previous studies reported in the literature. The review of English literature available suggests that most of the studies report almost equal to but slightly better accuracy rates with the robotic screw placement. The overall distinction of either of the two techniques is still debatable. Outcomes largely depend on the experience of the surgeon, as well as, the number of surgeons performing the robotic surgery technique in the particular study. Moreover, all these studies were single-center studies sharing their personal experiences. A larger number of surgeons add variability, while having fewer surgeons in the study can skew the results. Future multicenter studies with larger sample sizes and more surgeon participation are emphasized.

Since minor deviations rarely become symptomatic, many clinicians accept deviations up to 2 or 3 mm. In a meta-analysis of 4,790 screws conducted by Lonstein, et al. A total of 5.1% screws were reported to breach the cortical bone and only 0.2% of these caused neurological symptoms [29]. However, surgeons will be confronted with remaining or new-onset symptoms in the presence of a minor screw deviation and face the dilemma whether to re-operate or not.

The comparative results for operative time and hospital stay between the two groups were statistically insignificant (p = 0.192 and p = 0.587, respectively) in our study and were consistent with the earlier studies [3, 23]. Kantelhardt, et al. reported a better duration of postoperative hospitalization, postoperative opioid administration (for pain), infection rate and rate of screw revisions in their robotic guided group which is similar to the results reported by Schatlo, et al. [3, 26]. We found blood loss to be less in robotic as compared to hand placed which coincides with the findings of Schatlo, et al. [3].

The choice of approach between robotic versus the fluoroscopy-guided or CT stealth-guided hand-placed screws is more on the preference of the operating surgeon since there is no significant difference in either of the methods in terms of accuracy and patient safety. In fact, there would be hesitation to strongly rely on the results of this trial due to the limitations of low sample size. The main limitation of this study is low sample size. Although only six patients, there were a large number of pedicle screws to examine. The reason for low patient volume was due to the institution not having regular postoperative CT scans, which severely limited the number of patients included in this study. These factors will be taken into account on the upcoming multicenter prospective study.

This study was a preliminary pilot study for the development of a future study of pedicle screw accuracy assessment. The upcoming trial with a much larger cohort and large number of participating surgeons will have improved study protocol which will examine two groups once again but instead of robotic versus other types of screw placement, it will be CT or stealth-guided placement versus placement with fluoroscopy. Fluoroscopic placement is when the surgeon relies on fluoroscopic X-Ray for the location or trajectory of the screws. A study like that would provide readers with information on whether advancements in the real-time image technology will show better accuracy which is predicted. This pilot study was able to provide practice and education to better conduct the upcoming study.

## Conclusions

Our findings agree with most prior studies that there was no statistically significant difference in the accuracy of pedicle screw placement between the two methods of screw placement. Therefore, the techniques are equivocal in accuracy. The new technology (robotic-guidance) is as safe as conventional fluoroscopy assisted techniques and CT-guided stealth techniques for screw placement, but does not supersede the conventional free-hand method, though the literature seems to be more optimistic. Just like in any surgery, the technique preference should remain surgeon dependent. It should be emphasized that the study was a pilot study to learn how to conduct future studies on pedicle screw placement. The results from this study cannot hold a strong conclusion from the results due to low sample size. In the future study, CT stealth guided and robotically guided screw placements will be compared with fluoroscopy guided placement of screws for accuracy. The increase in sample size will be obtained by regularly performing postoperative CT scans on patients with thoracolumbar-sacral pedicle screw instrumentation and making it a multicenter study to involve more surgeons to eliminate any result bias.
